# Evaluating the Balloon-Assisted Rapid Intermittent Sequential Coiling Technique for the Treatment of Wide-Neck Cerebral Aneurysms

**DOI:** 10.7759/cureus.76885

**Published:** 2025-01-03

**Authors:** Osama Awad, Mohammad Al-Shatouri, Magdy El-Nisr, Ahmed Elbassiouny, Mohamed Habba

**Affiliations:** 1 Department of Diagnostic and Interventional Radiology, Suez Canal University Hospital, Ismailia, EGY; 2 Department of Neurology, Ain Shams University, Cairo, EGY

**Keywords:** balloon-assisted coiling, coil prolapse, endovascular treatment, neurointerventional procedure, subarachnoid hemorrhage, wide cerebral aneurysms

## Abstract

Background: Balloon-assisted coiling (BAC) is acknowledged as an auxiliary method for the endovascular treatment of difficult wide-necked cerebral aneurysms (WNCAs). An intracranial stent may be necessary as a supportive scaffold when the anatomical conditions are unfavorable, as BAC alone may be inadequate to avoid coil protrusion into the parent artery. We aimed to evaluate the safety of the balloon-assisted rapid intermittent sequential coiling technique (BARISCT) and the effectiveness of BARISCT in reducing the risk of coil prolapse in the primary artery during the coiling of WNCA.

Methodology: From March 2021 to April 2023, a quasi-experimental investigation was conducted on more than 19 patients with WNCA who visited the Neurointervention Unit at Suez Canal University Hospital and fulfilled the inclusion criteria of WNCA, specifically defined by an unfavorable dome-to-neck ratio of less than 2 or a neck length exceeding 4 mm.

Results: BARISCT has proven to be a safe and successful tool for occluding ruptured and unruptured WNCA, with a full occlusion rate of roughly 73% with minimum sequelae and no major complications.

Conclusion: BARISCT seems to be a method that is both safe and successful for the endovascular treatment of wide-neck intracranial aneurysms (WNCA), with no concern regarding the potential for an increase in the likelihood of complications.

## Introduction

Intracranial aneurysms, which originate from medium-sized arteries, result from congenital and acquired media defects and exposure to factors that promote degenerative changes in the arterial wall, such as hypertension. The reported frequency of intracranial aneurysms in the autopsy series was highly variable, ranging from 0.2% to 9.9% of the population (mean frequency 5%). The aggregate prevalence for adults without risk factors was 2.3% [1].

The aggregate five-year rupture rates for aneurysms in the anterior circulation among patients devoid of a history of subarachnoid hemorrhage were as follows: the proportion of individuals with aneurysms measuring less than 7 mm is 0%, 2.6% for those ranging from 7 to 12 mm, 14.5% for those between 13 and 24 mm, and 40% for those 25 mm or above. These statistics underscore the increasing risk associated with larger aneurysms [1].

For similar size categories, the rupture rates of posterior circulation aneurysms, including those of the posterior communicating artery (PCom), were 2.5%, 14.5%, 18.4%, and 50%, respectively. For patients with a previous history of subarachnoid hemorrhage, the cumulative rupture rate for five years for anterior circulation aneurysms that were smaller than 7 mm was 1.5%, but the rate for posterior circulation aneurysms of the same size was 3.4% [1].

Endovascular procedures for treating cerebral aneurysms have undergone substantial advancements ever since the Guglielmi detachable coils were introduced [2]. Endovascular procedures are particularly difficult for the treatment of complicated wide-necked cerebral aneurysms (WNCAs). However, balloon-assisted coiling (BAC) has gained acceptance as an adjunctive method for managing these intricate aneurysms. By providing better control and stability during coil placement, BAC improves the success rate of endovascular procedures in these challenging cases. An intracranial stent may be necessary as a supportive scaffold when the anatomical conditions are unfavorable, as BAC alone may be inadequate to avoid coil protrusion into the major arterial. In cases of aneurysmal subarachnoid hemorrhage, where antiplatelet medication before stent implantation may be prohibited, this could make matters more complicated [3-10].

We evaluated the effectiveness of the balloon-assisted rapid intermittent sequential coiling technique (BARISCT) in WNCA patients, particularly in cases with complex tortuous vascular anatomy or insufficient parent artery size for stent placement. This approach aimed to provide a viable alternative to conventional methods, ensuring better outcomes for these complex cases.

## Materials and methods

Study population

From March 2021 to April 2023, this quasi-experimental investigation was applied to more than 19 patients who presented to the Neurointervention Unit at Suez Canal University (SCU) Hospital in Ismailia with a WNCA.

This study received approval from the SCU's Medical Ethical Committee, registration number 4669/2021. The patient's caregivers provided written and informed assent and were thoroughly informed about the intervention outcomes. The patient was assured the right to engage in or exit the research at any time, without any consequences for their treatment or rapport with healthcare practitioners. Caregivers were kept informed about every step of the procedure, ensuring full transparency throughout the study.

Study procedures and data collection

Patients who were eligible for inclusion with WNCA were specifically characterized by a neck length greater than 4 mm or an unfavorable dome-to-neck ratio (DNR) of less than 2. Unruptured aneurysms were treated electively, and the decision to intervene was based on factors such as location, patient symptoms, and risk of rupture.

The exclusion criteria encompassed patients with contraindications to angiography, including those with allergic responses to contrast media or renal impairment that prohibits administering contrast agents. Additionally, patients with a favorable DNR, which suggests a lower risk of aneurysm complications, were also excluded from the study. This helps ensure the study focuses on the patient population most likely to benefit from the intervention being evaluated. All procedures were performed in a specialized neuroangiography facility (Allura Xper FD10, Philips Medical Systems, Philips, The Netherlands).

The primary researcher (O.A.) assisted a highly experienced neurointervention consultant with over 20 years of experience in the field, who conducted all procedures.

A femoral artery puncture was performed under sterile conditions. A 6F or 7F introducer sheath was placed in the femoral artery. Patients received an intravenous heparin bolus (usually 70-100 units/kg) after sheath placement to achieve an activated clotting time (ACT) of 250-300 seconds. A guiding catheter, such as the long sheath system, was used to advance to the appropriate cervical portion of the internal carotid artery or vertebral artery. During the procedure, continuous monitoring of ACT was performed, with additional heparin boluses administered as necessary.

For coil deployment, we utilized a microcatheter (Excelsior SL-10) incorporating thin-wall technology and a lubricious Hydrolene® coating (0.014 inch), along with a 0.012-inch guidewire (Stryker Neuroendovascular, Fremont, CA). For BAC, according to availability, we used compliant balloons (Eclipse, HyperGlide, TransForm, or HyperForm) along with a compatible guidewire, which was navigated to the parent vessel across the aneurysm neck.

These balloons were inflated across the aneurysm neck to serve as a temporary scaffold while coiling using Target® coils equipped with an electrolytic detachment mechanism (Stryker Neuroendovascular). Coils were deployed until the aneurysm sac was densely packed, ensuring sufficient occlusion. Heparin was discontinued postprocedure, and patients were monitored for groin complications or delayed bleeding.

Digital subtraction angiography is performed to assess coil placement and aneurysm occlusion. Noncontrast CT was performed for selected patients if indicated within 24 hours to rule out complications such as hemorrhage or ischemia.

Statistical analysis

The Statistical Package for the Social Sciences software, version 26 (IBM Corp., Armonk, NY), was employed for the statistical analysis of the collected data. The Kolmogorov-Smirnov test was employed to assess the normality of the data. Data were presented in tables and graphs when relevant. Frequencies and relative percentages were employed to illustrate qualitative data. The chi-square test (χ^2^) was employed to evaluate the association between qualitative factors, as previously indicated. The mean and standard deviation were employed to depict quantitative data.

## Results

Demographic data are detailed in Table 1.

**Table 1:** Sociodemographic data of the participants SD: standard deviation

Variable	n = 19
Age (years), mean ± SD	59.0 ± 12.5
Gender, n (%)	
Male	9 (47.4)
Female	10 (52.6)

Anterior cerebral artery was the most common location of the wide-neck intracranial aneurysms (WN-IAs; 42.1%, n = 8), followed by the middle cerebral artery (MCA; 31.5%, n = 6), then PCom (10.5%, n = 2), anterior choroidal artery (5.3%, n = 1), basilar artery (5.3%, n = 1), and ophthalmic artery (5.3%, n = 1) (Table 2).

**Table 2:** Location of the WN-IAs among the participants ACom: anterior communicating artery; MCA: middle cerebral artery; PCom: posterior communicating artery; WN-IAs: wide-neck intracranial aneurysms

Location	n = 19, n (%)
ACom	8 (42.1)
Anterior choroidal artery	1 (5.3)
Basilar artery	1 (5.3)
MCA	6 (31.5)
Ophthalmic artery	1 (5.3)
PCom	2 (10.5)

Fourteen patients (73.7%) had ruptured aneurysms, and five patients (26.3%) had unruptured aneurysms (Table 3).

**Table 3:** Incidence of aneurysmal rupture among study participants

Variable	n (%)
Ruptured	14 (73.7%)
Unruptured	5 (26.3%)
Total	19 (100%)

The mean dome diameter was 5.4 ± 1.3 mm, the mean neck diameter was 3.2 ± 0.9 mm, and the DNR was 1.7 ± 0.3 (Table 4).

**Table 4:** Dome and neck of IAs among the participants DNR: dome-to-neck ratio; SD: standard deviation; IAs: intracranial aneurysms

Variable	n = 19, mean ± SD
Dome (mm)	5.4 ± 1.3
Neck (mm)	3.2 ± 0.9
DNR	1.7 ± 0.3

The types of balloons used in the technique were Eclipse, HyperForm, HyperGlide, and TransForm balloons, which had a maximum inflation time of 5 minutes (Table 5).

**Table 5:** Balloon used among the participants

Balloon type	n = 19, n (%)
Eclipse	6 (31.6%)
HyperForm	4 (21.1%)
HyperGlide	4 (21.1%)
TransForm	5 (26.2%)

**Figure 1:**
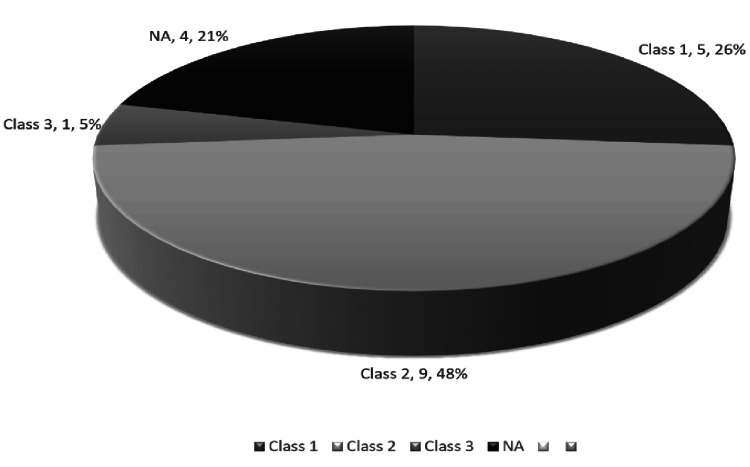
Modified Rankin scale at baseline among the participants NA: not applicable

Four patients (21.1%) were presented with headaches, 14 patients (73.7%) were presented with subarachnoid hemorrhage, and only one patient (5.3%) was discovered incidentally. Fourteen patients (73.7%) were categorized as class 1 according to the Raymond scale, while five patients (26.3%) were categorized as class 2. Nine patients (47.4%) were modified Rankin scale (mRS) 2 at baseline, five patients (26.3%) were mRS 1, and one patient (5.3%) was mRS 3 (Table 6 and Figure 1).

**Table 6:** Clinical presentation of the participants mRS: modified Rankin scale; NA: not applicable

Variable		n = 19, n (%)
Presentation	Headache	4 (21.1)
	Incidental finding: trauma survey	1 (5.3)
	Subarachnoid hemorrhage	14 (73.7)
Raymond scale	Class 1	14 (73.7)
	Class 2	5 (26.3)
mRS (baseline)	1	5 (26.3)
	2	9 (47.4)
	3	1 (5.3)
	NA	4 (21.1)

There was a statistically significant change between the mRS at the beginning of the study and after the patient was discharged (Table 7).

**Table 7:** Association between mRS baseline and mRS discharge Chi-square test was used ^*^p is significant at <0.05 mRS: modified Rankin scale; NA: not applicable

mRS baseline	mRS discharge			p value
	0	1	NA	
1	5	0	0	<0.001^*^
2	7	2	0	
3	0	1	0	
NA	0	0	4	

## Discussion

Traditional endovascular techniques, such as coiling, BAC, stent-assisted coiling, or flow diversion, can be challenging when treating complex anatomical aneurysms, such as WNCA. While endovascular therapy remains the primary treatment for most cerebral aneurysms, coiling can be difficult in these complex cases [11].

It can be challenging to maintain control over some types of aneurysms, including huge and giant aneurysms, fusiform aneurysms, large neck aneurysms, and those with an unfavorable size ratio between the aneurysm dome, the neck, and the parent artery. Image-guided endovascular therapy for cerebral aneurysms continues to face substantial limitations; these limitations include the hazards of coil protrusion into the parent artery, inadequate aneurysm occlusion, and aneurysm recanalization [12].

This resulted in the development of novel techniques and technologies, such as the BAC technique, which is also known as the remodeling technique, aneurysm coiling with stenting, and, more recently, flow diversion/disruption. Nonetheless, aneurysms characterized by complex morphology, unfavorable angulation with the parent artery, and large necks present a therapeutic challenge, exhibiting a significantly elevated risk of recurrence [13].

The balloon-assisted method involves inserting a balloon that cannot be detached into the parent artery and then inflating it to block the neck of the aneurysm. This helps to support the microcatheter while it is being deployed and prevents the coil from impinging on the parent artery. In contrast to stents, the balloon does not remain in place within a cerebral conduit for an extended time. In addition, no requirement for antiplatelet therapy or pharmaceutical therapy is protracted [14].

This quasi-experimental investigation was performed at the Neurointervention Unit of SCU Hospital in Ismailia, Egypt. This research aimed to examine the safety and efficacy of BARISCT in lowering the risk of coil prolapses in the parent artery during the coiling of WNCA. This study included 19 patients with WN-IAs, with a mean age of 59.0 ± 12.5 years. Female patients made up 52.6% (n = 10) of the sample.

**Figure 2:**
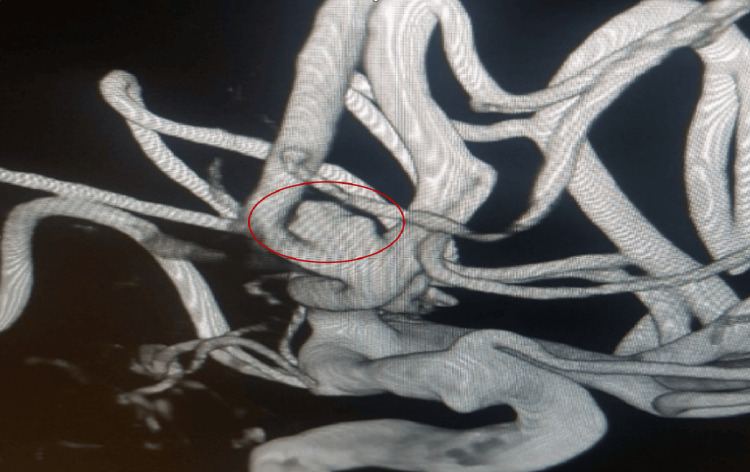
Three-dimensional reformatted angiography showing wide-neck ACom aneurysm Subarachnoid hemorrhage was present in a patient who was 69 years old. Three-dimensional reformatted angiography revealed a wide-neck ACom aneurysm that measured 5.8 mm at the dome and 4 mm at the neck. The DNR for this aneurysm was 1.45 ACom: anterior communicating artery; DNR: dome-to-neck ratio

In the current research, anterior communicating artery (ACom) was the most common location of the WN-IAs (42.1%, n = 8), as shown in Figure 2, followed by MCA (31.5%, n = 6), PCom (10.5%, n = 2), anterior choroidal artery (5.3%, n = 1), basilar artery (5.3%, n = 1), and ophthalmic artery (5.3%, n = 1). Similarly, intracranial aneurysms were common in ACom (40.3%), MCA (20.3%), and only 7.1% in the basilar artery [15]. This corresponds to the study by Mihalea et al., which identified an increased occurrence of aneurysms in the middle cerebral and ACom [16]. Aneurysm locations were ACom (36%), MCA (41.9%), and basilar artery (8.1%) [17]. In the series by Altay et al., MCA and ACom had more aneurysms (30% and 19.2%) among patients who underwent modified BAC embolization [18]. Additionally, it was found that 25.5% of cerebral aneurysms were in ACom, 28.3% in MCA, and 11.7% in PCom [19]. Dabus et al. agreed that aneurysms were found in ACom among 34.5% and MCA among 6% [20].

A cohort research was done to evaluate the angiographic and clinical results of aneurysms treated with BAC embolization compared to those treated with unaided coil embolization in the ruptured aneurysm cohort. The average age of patients was comparable to this study, at 58.7 ± 12.1 years, with female patients constituting 71.4% of those treated with BAC embolization [20].

The current study agreed that 21.1% (n = 4) presented with headaches, 73.7% (n = 14) presented with subarachnoid hemorrhage, and only 5.3% (n = 1) were discovered incidentally. Consistent with these findings, approximately 91.9% of the individuals experienced a subarachnoid hemorrhage, 5.4% exhibited unruptured aneurysms, and 2.7% manifested cerebral hemorrhage [14]. The majority of unruptured aneurysms are asymptomatic and identified accidentally; nonetheless, aneurysm rupture leads to a potentially fatal subarachnoid hemorrhage [21].

A study was conducted in which the average diameter of the dome was 5.4 ± 1.3 mm, the neck diameter was 3.2 ± 0.9 mm, and the ratio of the dome to the neck was 1.7 ± 0.3. The study by Metwaly et al. showed an average aneurysm diameter of 5.7 ± 1.7 mm and a mean neck diameter of 3.8 ± 1.0 mm [14]. Pierot et al. concurred that the majority of patients (70.4%) exhibited an aneurysm diameter of 5 mm or more, whereas 73.2% presented a neck diameter of 4 mm or less [15].

Invariably, individuals with cerebral aneurysms had an average dome width of 6.55 mm, a mean neck size of 4.5 mm, and a mean DNR of 1:1.46 [16]. The dome diameter was 6.7 ± 2.6 mm, the neck diameter measured 3.2 ± 1.2 mm, and the DNR was 1.6 ± 0.6 [20]. The neck measured 4.32 ± 1.66 mm, the greatest diameter was 6.89 ± 2.45 mm, and the DNR was 1.35 ± 0.26 [18].

The maximum and neck diameters in patients with cerebral aneurysms were 6.1 ± 3.1 and 3.1 ± 1.3 mm, respectively [19]. The neck diameter reported was lower than what was mentioned in these results, and this is because Wallace et al. studied patients with cerebral aneurysms, and only 77.5% of them had wide necks.

**Figure 3:**
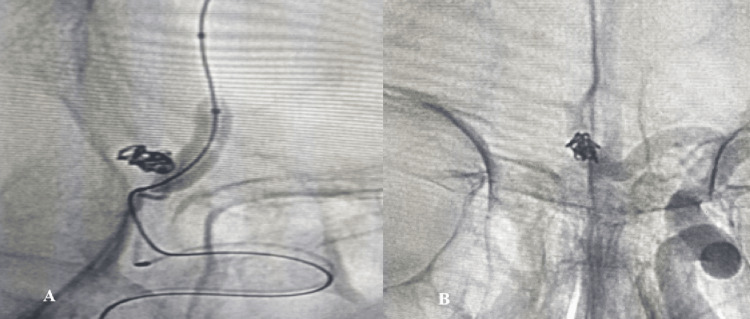
Sequential WNCA occlusion by BAC of the same patient. (A) BAC was selected to obstruct the WNCA. The intraprocedural angiography confirmed that the aneurysm was entirely occluded following the deployment of detachable coils. (B) The full blockage of a WNCA, categorized as Raymond class 1. This rating demonstrates the efficacy of the treatment strategy by indicating a successful surgery with no remaining aneurysm filling WNCA: wide-necked cerebral aneurysm; BAC: balloon-assisted coiling

**Figure 4:**
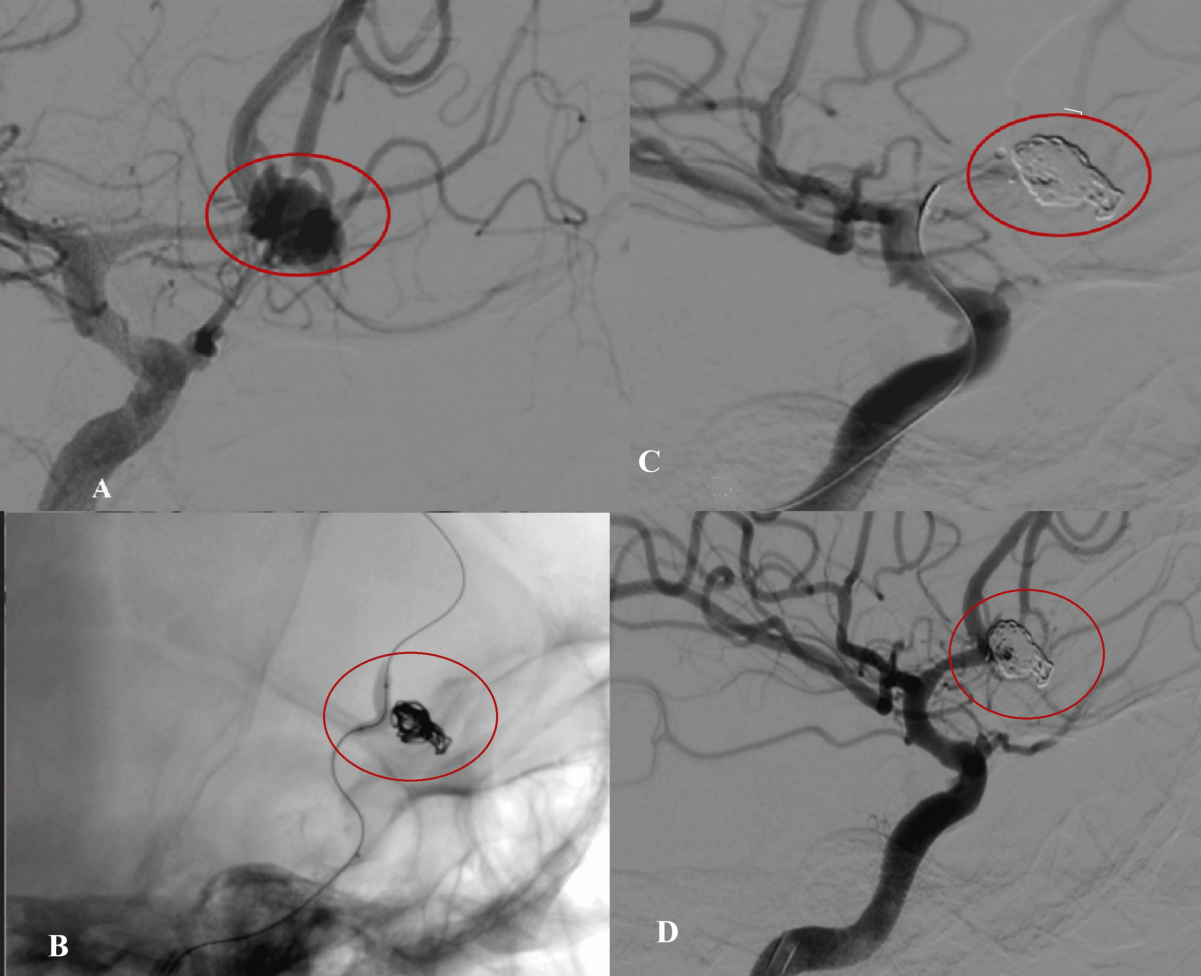
DSA of ACom WNCA (pre- and postocclusion by BAC). A 49-year-old woman presented with subarachnoid hemorrhage. (A) DSA was done and revealed an ACom wide neck aneurysm, measuring 4.97 mm at the dome and 3.04 mm at the neck; DNR was 1.6. (B) Balloon remodeling during the coiling of WNCA. (C,D) Occlusion of a WNCA by BARISCT, classified as Raymond class 1. This classification indicates a successful procedure with no residual aneurysm filling, showcasing the effectiveness of the treatment approach DSA: digital subtraction angiography; ACom: anterior communicating artery; WNCA: wide-neck intracranial aneurysms; BAC: balloon-assisted coiling; BARISCT: balloon-assisted rapid intermittent sequential coiling technique; DNR: dome-to-neck ratio

Based on the Raymond-Roy occlusion categorization, 73% were classified as class 1 (complete obliteration) by the Raymond scale, and 26.3% were class 2 (residual neck). Similarly, 86.5% were completely obliterated, 10.8% had a remnant neck, and 2.7% had a residual aneurysm [14]. Shapiro et al. established that the utilization of balloons correlated with enhanced initial and subsequent angiographic occlusion rates [22]. Dabus et al. [20] reported analogous findings in a prospective series, indicating that balloon remodeling achieved a statistically significant increase in instantaneous occlusion rates (64%). Class 1 occlusion was detected in 631 (73%) of 864 aneurysms, class 2 occlusion was found in 176 (20.4%), and class 3 occlusion was found in 57 (6.6%) of the aneurysms, according to those who participated in a parallel study that analyzed 800 occurrences [23]. Figures 3, 4 show BAC of WNCA with class 1 occlusion.

The immediate postembolization angiographic results of a group of 34 aneurysms that were treated with the remodeling technique using the HyperForm balloon revealed entire occlusion in 31 cases or 91.2% of the cases, and partial occlusion in three cases or 8.8% of the cases [24].

The results of 52 aneurysms in 50 patients were published by Moret et al. in their preliminary report on the therapeutic use of the balloon-assisted procedure in humans. They achieved complete occlusion in 77% of instances, subtotal occlusion in 17% of cases, and incomplete occlusion in 6% of cases [25]. Sedat et al. showed that stenting and coiling resulted in long-term full aneurysmal closure in 71% of the WNCA [26].

The balloon used in the technique was an Eclipse in 31.6% (n = 4), HyperForm in 21.1% (n = 3), and HyperGlide in 21.1% (n = 3), while a transform balloon was used in 26.2% (n = 2). There were 48.6% of cases in which Metwaly et al. used HyperForm balloons, 43.2% of cases used HyperGlide balloons, 5.4% of cases involved Copernic balloons, and 2.7% of cases involved Eclipse balloons [14]. Meanwhile, HyperGlide was used in 50% of aneurysms [16].

This study found that nine patients (47.4%) were mRS 2 at baseline, 26.3% were mRS 1, and 5.3% were mRS 3. At discharge, mRS was 0 among 63.2% and 1 among 15.8%. The mRS at baseline and discharge exhibited a statistically significant difference. All patients exhibited an mRS score of 0 at the time of discharge [16]. At discharge, 71 patients exhibited neurological impairments, representing 8.9% of the overall population. Of the 71 patients, 36 recovered well with an mRS score of less than 2, whereas 35 had an mRS score of 2 or above, accounting for 4.4% of the whole cohort three to six months after the end of treatment [23]. In agreement, following treatment with a HyperForm balloon by the BAC, 15 patients with unruptured aneurysms returned to their prior employment and remained asymptomatic (mRS 0) [24].

This study agreed that only one case showed arterial spasm, while 94.7% (n = 18) showed no postintervention complications. This goes in line with Metwaly et al.; the balloon was hyperinflated for five minutes until the hemorrhage ceased, and 2.7% of the patients experienced ischemia, while 5.4% experienced mild rupture by wire. Thus, nearly 91.9% of the patients experienced no complications [14]. Similar results were obtained from the literature; no hemorrhagic or thrombotic complications occurred postintervention [16]. Altay et al. agreed that for patients who underwent BAC embolization, only one patient (3.8%) showed thromboembolism [18].

Along the same line, the remodeling technique with the HyperForm balloon did not result in any mortality caused by subarachnoid hemorrhage or complications during or after treatment [24]. Unlike these results, 39.3% of the patients exhibited coil herniation, 7.5% thrombosis on the coil ball, and 21.3% packing density [20].

Nonetheless, others claim that this strategy increased procedure morbidity despite the positive outcomes [27]. In contrast, developments in coil technology (such as enhanced framing capabilities with three-dimensional, 360° designs) have been made [28], and numerous coiling methods, including the double microcatheter technique [29], reduced the use of balloons as supplemental instrumentation.

Sluzewski et al. found that procedure-related complications that resulted in death or dependence were much higher in BAC embolization (14.1%) than in coil embolization (3%). This was reported in a cohort of 827 endovascularly treated aneurysms. The authors discovered a noteworthy pattern that demonstrated an increased retreatment rate in aneurysms treated using BAC embolization [27].

A study comparing the clinical and radiologic outcomes of WNCA treated with stent-assisted coil embolization, double catheter, and BAC embolization discovered no differences in recurrence or periprocedural complication rates, as well as no incidences of rebleeding or aneurysmal rupture following therapy. Although the stent-assisted coil embolization and balloon-assisted coil embolization groups had somewhat greater complete occlusion rates (63.8% and 63.2%, respectively) than the twin catheter group (46.7%), there were no cases of rebleeding or aneurysmal rupture following treatment [29].

Limitations

Despite the encouraging angiographic results on the patients, drawing conclusions regarding recurrence and recanalization based on this patient’s number and the absence of follow-ups are limitations of this study. A larger, controlled study is planned to evaluate BARISCT and overcome these limitations.

## Conclusions

According to this study, BARISCT is considered a secure and efficacious method for the endovascular treatment of WNCA, with no concerns regarding a possible increase in complication rates. It enhances both the safety of the procedure and the efficacy of the endovascular treatment. This method has the significant technical advantage of achieving complete and stable occlusions, which is the primary objective of endovascular therapy. This method demonstrated little issues and favorable results.
